# West Nile Virus in Horses, Guatemala

**DOI:** 10.3201/eid1206.051615

**Published:** 2006-06

**Authors:** Maria Eugenia Morales-Betoulle, Herber Morales, Bradley J. Blitvich, Ann M. Powers, E. Ann Davis, Robert Klein, Celia Cordón-Rosales

**Affiliations:** * Universidad del Valle de Guatemala, Guatemala City, Guatemala;; † Ministry of Agriculture and Livestock, Guatemala City, Guatemala;; ‡ Colorado State University, Fort Collins, Colorado, USA;; § Centers for Disease Control and Prevention, Fort Collins, Colorado, USA;; ¶ US Department of Agriculture-Animal and Plant Health Inspection Service, Guatemala City, Guatemala

**Keywords:** West Nile virus, horses, Guatemala, letter

**To the Editor:** West Nile virus (WNV, *Flaviviridae*: *Flavivirus*) is emerging as a public health and veterinary concern. Since its introduction into North America in 1999, it has spread rapidly, reaching the Caribbean Basin in 2001, Mexico in 2002, El Salvador in 2003, and Colombia in 2004 (*1*). However, reports of equine illness and deaths in Latin America are inconclusive. With the exception of viral isolates from a dead bird, a human, and a mosquito pool in Mexico (*2**,**3*), all reports of WNV presence in Latin America have relied on serologic evidence. WNV is a member of the Japanese encephalitis serocomplex, which in the Western Hemisphere includes St. Louis encephalitis virus (SLEV) (*4*). Serologic investigations for WNV in Latin America must use highly specific assays to differentiate WNV infection from potentially cross-reactive viruses such as SLEV or possibly additional unknown viruses. In particular, SLEV is of concern since it was previously isolated from Guatemalan mosquitoes (*5*).

Alerted by the findings of WNV transmission in the region (*1*), we collected serum samples from horses from 19 departments of Guatemala from September 2003 to March 2004, to initially estimate the extent of WNV spread and its potential public health risk. Because no animals exhibited signs of neurologic illness at the time of the survey, only healthy horses were sampled. Before 2005, equine WNV vaccines were prohibited and unavailable in Guatemala (Unidad de Normas y Regulaciones, Ministerio de Agricultura Ganadería y Alimentación, Guatemala, pers. comm.); as such, cross-reactivity due to prior vaccination is highly unlikely. Samples were initially tested for WNV-reactive antibodies by using an epitope-blocking enzyme-linked immunosorbent assay (blocking ELISA) (*6*). The ability of the test sera to block the binding of the monoclonal antibodies to WNV antigen was compared to the blocking ability of control horse serum without antibody to WNV. Data were expressed as relative percentages and inhibition values >30% were considered to indicate the presence of viral antibodies.

A subset of positive samples was further confirmed by plaque-reduction neutralization test (*7*). Of 352 samples, 149 (42.3%) tested positive with the 3.1112G WNV-specific monoclonal antibody. Of 70 blocking ELISA–positive samples, the neutralization tests indicated the infecting agent was WNV, SLEV, and undifferentiated flavivirus in 9, 33, and 21 samples, respectively. Titers were expressed as the reciprocal of serum dilutions yielding >90% reduction in the number of plaques in a plaque-reduction neutralization test (PRNT_90_). PRNT_90_ titers of horses seropositive for WNV ranged from 80 to 320. PRNT_90_ titers of horses seropositive for SLEV ranged from 40 to 2,560. For the differential diagnosis of samples with neutralizing antibody titers against both WNV and SLEV in this test, a >4-fold titer difference was used to identify the etiologic agent. The undifferentiated flavivirus-reactive specimens had <4-fold difference in cross-neutralization titers. Likely possibilities for the inability to distinguish the infecting virus include previous infection with these or other flaviviruses (previously described or unknown) resulting in elevated cross-reactive titers. The remaining 10% of specimens that tested negative by PRNT probably represent nonneutralizing antibodies in the serum or false positivity in the blocking ELISA.

Our serologic results provide indirect evidence of past transmission of WNV, SLEV, and possibly other flaviviruses to horses in Guatemala. Although no confirmed cases of WNV-attributed disease have been reported in Central America to date, flavivirus transmission appears to be widely distributed in Guatemala (Figure). Efforts are under way to confirm WNV transmission by viral isolation and to evaluate the impact of WNV on human, horse, and wildlife populations. More information is needed to establish the public health threat of WNV and other zoonotic flaviviruses in the region.

**Figure:**
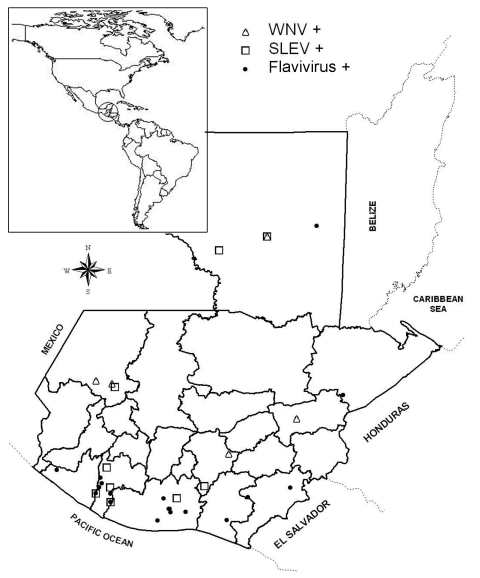
Geographic distribution in Guatemala of horses showing previous infections with West Nile virus (WNV), Saint Louis encephalitis virus (SLEV), or undifferentiated flavivirus as confirmed by plaque reduction neutralization test. Each location may have multiple positive horses.
